# Prevalence and Risk Factors of Discordance Between Hip and Spinal Bone Mineral Density Among Saudi Subjects

**DOI:** 10.7759/cureus.27684

**Published:** 2022-08-04

**Authors:** Homoud Al Zaid, Muhannad S Alamri, Abdulhadi A AlOfair, Faisal S Alqusaiyer, Adel I Alorainey, Mohammad I Alasqah, Riad A Sulimani

**Affiliations:** 1 Department of Internal Medicine, College of Medicine, King Saud University, Riyadh, SAU; 2 Department of Medicine, King Saud University, Riyadh, SAU

**Keywords:** saudi arabia, obesity, hip spine discordance, dual x ray absorptimetry, s: osteoporosis

## Abstract

Background

Discordance between hip and spine on dual-energy x-ray absorptiometry is a well-known problem in diagnosing osteoporosis. The prevalence and risk factors of this problem have not been studied in the Saudi population. The objective of this study was to document this discordance in our population and its possible risk factors.

Materials and methods

We analyzed data obtained from subjects who had dual x-ray absorptiometry (DXA) between January 2021 and December 2021 at King Khalid University Hospital, Riyadh, Saudi Arabia. Subjects with the following conditions were excluded: secondary osteoporosis, patients taking anti-osteoporotic agents, patients on steroids or hormonal replacement therapy, hyperparathyroidism, hypoparathyroidism, and chronic renal disease. A total of 1388 patients satisfied our inclusion criteria. World Health Organization (WHO) criteria for diagnosis were implemented. Major discordance was defined as osteoporosis in one site and normal in the other. Minor discordance was defined as a difference of no more than one World Health Organization diagnostic class between two sites. Bivariate statistical analysis was achieved using appropriate statistical tests (chi-square, student’s t-test, one-way analysis of variance, and Pearson’s correlation), based on the type of study and outcome variables. A p-value of < 0.05 and 95% CI were used to report the statistical significance and precision of results.

Results

A total of 1388 subjects were analyzed, of which, 1196 (86%) were females with a mean age of 58.8 (13.8 SD) and 192 were males with a mean age of 58 (18.0 SD). Lumbar osteoporosis was found in 312 (22.5%) participants while hip osteoporosis was reported in 73 (5.3%) of the participants. Major discordance was documented in 85 (6.1%) of all participants (6.3% of the male and 6.1% of the female patients). All of these subjects had lumbar spine osteoporosis with normal hip bone mineral density (BMD). Minor discordance was found in 591 patients (42.6%). Obesity (BMI > 30) was found to be a risk factor for both major (2.10-11.6, 95% CI) and minor (2.5-11.4, 95% CI) discordance.

Conclusion

Discordance between hip and spine BMD is common among Saudi subjects. Lumbar spine osteoporosis with normal hip BMD caused this discordance in our subjects. Obesity could be responsible for the occurrence of this discordance. Mechanisms may include higher rate of turnover in spine, technical artifacts in the measurements of lumbar spine BMD, or due to the effects of weight loading. Caution should be exercised when interpreting DXA results, especially in obese subjects.

## Introduction

The assessment of bone mineral density (BMD) and measurement of T-scores using a dual-energy x-ray absorptiometry (DXA) scan are valuable in diagnosing osteoporosis (OP) and the individual’s risk of developing fragility fractures [1,2]. A DXA scan is performed by measuring BMD at the lumbar spine (L1-L4) and total femoral head. Based on the World Health Organization (WHO) criteria, the DXA scan result can categorize the individual’s bone density status as normal, osteopenic, or osteoporotic according to the T-scores [3]. Although the hip and spine are expected to have consistent T-scores, it has often been noted that both sites can display different diagnostic categories [4]. Major discordance is marked by the presence of different T-scores, one suggesting osteoporosis at one skeletal site and another indicating normal BMD at the other skeletal site [5]. Minor discordance is defined by either having one skeletal site classified as normal, while the other site has osteopenia or one skeletal site exhibiting osteopenia, while the other site exhibits osteoporosis [5]. If the T-scores of both sites have the same diagnostic classification, this will indicate the presence of T-score concordance.

Despite the high prevalence of osteoporosis in Saudi Arabia, the prevalence of spine-hip discordance (SHD) in the Saudi Arabian population and its possible causes have never been documented. Thus, this study aimed to document the prevalence and possible causes of SHD in this population.

## Materials and methods

A retrospective cross-sectional study was conducted at King Khalid University Hospital, Riyadh, Saudi Arabia, after obtaining IRB approval. The data of all patients who had BMD between January 2021 and December 2021 were reviewed by the nuclear medicine department. The collected data were obtained from the patients’ medical records through the Electronic System for Integrated Health Information Software (E-SIHI; Riyadh, Saudi Arabia: King Saud University) used in the authors’ institute. The study included all female and male patients who underwent a DXA scan of the total hip and posterior-anterior lumbar spine (L1-L4). None of the patients had vertebral fracture assessment done. The following patients were excluded: 654 taking anti-osteoporotic agents, 36 diagnosed with secondary osteoporosis, 237 on steroids, 46 on hormonal replacement therapy, 72 on an anti-convulsant agent, 56 with hyperparathyroidism, and two with hypoparathyroidism, and 56 with possible chronic renal disease (serum creatinine greater than 120 μmol/L in women and 140 μmol/L in men and glomerular filtration rate {GFR} was not available in the patients studied). Further, 250 of the patients had incomplete medical records.

Out of 2793 patients, only 1388 patients satisfied our inclusion criteria. Each patient was categorized as having any of the following: concordance in osteoporosis, osteopenia, or normal T-scores on both sites, minor discordance (osteoporotic in one skeletal site and osteopenic in the other site, or osteopenic in one skeletal site and normal in the other site), or major discordance (osteoporotic in one skeletal site while the other site is in the normal range). The diagnosis of low BMD was established according to the WHO criteria [3]. The BMD and T-score of the total hip and lumbar spine (L1-L4) were measured by the Hologic Discovery Wi DEXA (Marlborough, MA: Hologic, Inc.). Osteoporosis was defined when bone mineral density is less than or equal to 2.5 standard deviations below that of young healthy women. Osteopenia was defined as a T-score -1.0 to < -2.5 below SD. T-scores ≤ -1.0 below SD were considered normal. There were 377 patients with normal T-scores, 778 with osteopenia, and 233 with osteoporosis.

Data analysis was performed using the SPSS statistical software package version 24 (Armonk, NY: SPSS Inc.). Descriptive statistics (mean, SD, frequencies, and percentages) were used to describe the quantitative and categorical variables. Bivariate statistical analysis was carried out using the appropriate statistical tests (chi-square, student’s t-test, one-way analysis of variance, and Pearson’s correlation) based on the type of study and outcome variables. A p-value of < 0.05 and 95% CI was used to report the statistical significance and precision of results.

## Results

Table 1 shows the demographic data of patients. Out of 1388 participants, 1196 (86%) were females with a mean age of 58.8 years (13.8 SD), and 192 were males with a mean age of 58 years (18.0 SD).

**Table 1:** Clinical characteristics of participants. Data were presented as mean±SD for numerical variables and frequency (%) for categorical variables. LS: lumbar spine; BMD: bone mineral density; FN: femoral neck

Toto	Total	Males	Females
N	1388	192	1196
Age	58.8±13.8	58.0±18.0	58.9±12.9
Height (cm)	159.7±7.4	170.8±7.2	157.9±5.7
Weight (kg)	74.1±15.9	72.3±19.6	74.4±15.2
BMI (kg/m^2^)	29.2±6.8	24.9±7.3	29.9±6.5
LS BMD	1.01±0.2	1.01±0.2	1.01±0.2
LS T-score	-1.54±1.3	-1.6±1.3	-1.5±1.3
BMD FN left	0.93±0.2	0.93±0.2	0.93±0.2
BMD FN right	0.92±0.4	0.97±1.0	0.92±0.2
FN left T-score	-72±1.2	-0.78±1.2	-0.71±1.2
FN right T-score	-0.79±1.2	-0.82±1.2	-0.78±1.2
Total hip T-score	-0.75±1.1	-0.80±1.1	-0.75±1.1
History of osteoporosis fracture	86 (6.2)	14 (7.3)	72 (6.0)

Table 2 shows the mean lumbar spine T-score was -1.6 (1.3 SD) among males and -1.5 (1.3 SD) among females, while the mean femoral neck T-score was -0.8 (1.1 SD) among males and -0.75 (1.1 SD) among females. Table 2 shows the characteristics of the participants; 312 (22.5%) participants had lumbar osteoporosis, where 265 (22.2%) were females and 47 (24.5%) were males. Hip osteoporosis was reported in 73 (5.3%) of the participants: 66 (5.5%) females and seven (3.6%) males.

**Table 2:** Classification of T-scores according to the WHO criteria in the lumbar spine and total hip. Data were presented as n (%) and 95% CI.

Diagnosis	Males (n=192)				Females (n=1196)				Total (n=1388)			
	Lumbar spine		Total hip		Lumbar spine		Total hip		Lumbar spine		Total hip	
	N (%)	95% CI	N (%)	95% CI	N (%)	95% CI	N (%)	95% CI	N (%)	95% CI	N (%)	95% CI
Normal	62 (32.3)	25.7-39.4	105 (54.7)	47.4-62	395 (33.0)	30.4-35.8	702 (58.7)	55.8-61	457 (32.9)	30.5-35.5	807 (58.1)	55.5-61
Osteopenia (T-score -1 to -2.5)	83 (43.2)	36.1-50.6	80 (41.7)	34.6-49	536 (44.8)	42.0-47.7	428 (35.8)	33.1-38.6	619 (44.6)	42.0-47.3	508 (36.6)	34.1-39.2
Osteoporosis (T-score ≤ 2.5)	47 (24.5)	18.6-31.2	7 (3.6)	1.5-7.4	265 (22.2)	19.8-24.6	66 (5.5)	4.3-7.0	312 (22.5)	20.3-24.8	73 (5.3)	4.1-6.6

Table 3 indicates that major discordance was found in 85 (6.1%) of all participants (6.3% of the male patients and 6.1% of the female patients); it was only noted in cases with osteoporosis of the lumbar spine and normal hip BMD. Minor discordance was found in 591 patients (42.6%). The majority of these cases displayed osteopenia of the lumbar spine and normal hip BMD.

**Table 3:** Distribution of diagnostic discordances using WHO criteria according to age and gender. Data were presented as n (%).

Classification	Age and gender effect distribution								Distribution of diagnostic discordance and T-score concordance		
	< 50 year		50-60 year		60-70 year		Above > 70 year				
	Males	Females	Males	Females	Males	Females	Males	Females	Males	Females	Total
Major T-score discordance	4 (6.6)	10 (4.6)	1 (3.4)	29 (8.5)	2 (4.6)	27 (6.1)	5 (8.6)	7 (3.5)	12 (6.3)	73 (6.3)	85 (6.1)
Hip osteoporosis, normal lumbar	0 (0.0)	0 (0.0)	0 (0.0)	0 (0.0)	0 (0.0)	0 (0.0)	0 (0.0)	0 (0.0)	0 (0.0)	0 (0.0)	0 (0.0)
Hip normal, lumbar osteoporosis	4 (6.6)	10 (4.6)	1 (3.4)	29 (8.5)	2 (4.6)	27 (6.2)	5 (8.6)	7 (3.5)	12 (6.3)	73 (6.3)	85 (6.1)
Minor T-score discordance	21 (34.4)	94 (43.1)	13 (44.8)	144 (42.2)	21 (47.7)	188 (42.8)	22 (37.9)	88 (44.5)	77 (40.1)	514 (42.8)	591 (42.6)
Hip osteoporosis lumbar osteopenia	0 (0.0)	2 (0.9)	0 (0.0)	1 (0.3)	0 (0.0)	2 (0.5)	0 (0.0)	1 (0.5)	0 (0.0)	6 (0.5)	6 (0.4)
Hip osteopenia, lumbar osteoporosis	10 (16.4)	19 (8.7)	3 (10.4)	41 (12.0)	9 (20.5)	47 (10.7)	6 (10.3)	25 (12.6)	28 (14.6)	132 (11.0)	160 (11.5)
Hip osteopenia, normal lumbar	1 (1.6)	9 (4.1)	1 (3.4)	19 (5.6)	3 (6.8)	25 (5.5)	4 (6.9)	18 (9.1)	9 (4.7)	71 (5.8)	80 (5.8)
Hip normal, lumbar osteopenia	10 (16.4)	64 (29.4)	9 (31.0)	83 (24.3)	9 (20.5)	114 (25.7)	12 (20.7)	44 (22.2)	40 (20.8)	305 (25.5)	345 (24.9)
T-score concordance	36 (59.0)	114 (52.3)	15 (51.8)	168 (49.3)	21 (47.7)	224 (51.1)	31 (53.5)	103 (52.0)	103 (53.6)	609 (50.9)	712 (51.3)
Hip and lumbar osteoporosis	3 (4.9)	11 (5.0)	3 (10.4)	11 (3.2)	0 (0.0)	24 (5.5)	1 (1.7)	14 (7.1)	7 (3.6)	60 (5.0)	67 (4.8)
Hip and lumbar osteopenia	16 (26.2)	38 (17.3)	7 (24.1)	70 (20.5)	10 (22.7)	78 (17.8)	10 (17.3)	39 (19.7)	43 (22.4)	225 (18.8)	268 (19.3)
Hip and lumbar normal	17 (27.9)	65 (30.0)	5 (17.3)	87 (25.6)	11 (25.0)	122 (27.8)	20 (34.5)	50 (25.3)	53 (27.6)	324 (27.1)	377 (27.2)
Total	61	218	29	341	44	439	58	198	192	1196	1388

Table 4 shows a significant correlation between obesity (BMI > 30) and both major (2.10-11.6, 95% CI) and minor (2.5-11.4, 95% CI) discordance. Gender and aging beyond 60 years did not have any effect on the occurrence of BMD site discordance.

**Table 4:** Multinomial logistic regression (MLR) analysis for risk factors of major and minor discordance with T-score concordance at lumbar and femoral sites as reference. *P-value significant at 0.01 level. Data were presented as odds ratio (95% CI).

Parameters	Reference T-score concordance osteoporosis	
	Major	Minor
Gender (female)	0.71 (0.26-1.92)	0.78 (0.34-1.77)
Age > 50	1.23 (0.55-2.77)	1.06 (0.57-1.97)
Age > 60	0.68 (0.41-1.3)	0.81 (0.50-1.4)
Age > 70	0.57 (0.25-1.3)	0.79 (0.4-1.5)
Obesity (> 30)	4.92 (2.10-11.6)*	5.33 (2.5-11.4)*
Hip fractures	2.42 (0.3-23.8)	5.96 (0.81-43.9)
Menopausal (female)	1.41 (0.6-3.6)	1.04 (0.5-2.1)

## Discussion

This study revealed that, in our sample population, there was a 6.1% prevalence of major discordance and 42.6% prevalence of minor discordance. The prevalence of major discordance in our cohort is higher than that in Malaysia 2.3%, Iran 2.7%, Morocco 4%, and India 3.35% [5-9]. However, it is lower than that of another study from India, which reported a major discordance of 16.67% [10]. The prevalence of minor discordance was also higher than in these countries. The rate of concordance was 51.3%, which is lower than some previous studies. It is interesting that all cases of major and minor discordance were related to lower spine BMD compared to FN, and not vice versa. Obesity was found to be responsible for the discordance, while, contrary to previous studies, increasing age was not. Indeed, the relationship between obesity and BMD is rather complex [11]. Conflicting data have emerged on the obesity-BMD relationship. The mechanical loading effect caused by excess adipose tissue has always been considered protective to bone [12,13]. However, it was noted that higher BMD in the femoral neck (FN) - but not the lumbar spine - was seen in overweight postmenopausal women when compared to a lean group [14]. The FN BMD also correlated with BMI, whereas this was not noted with lumbar spine BMD [14]. It was proposed that the effects of increased BMI on lumbar spine BMD in this regard are related to artifactual interference by abdominal fat [14,15]. Other than artifactual factors, waist circumference - a reflection of visceral fat - was noted to correlate with bone loss. The higher content of cancellous bone in the spine - in which higher bone turnover exists - was also suggested to explain the lower BMD of spine in women between 50 years and 60 years of age [16]. The majority of our patients were in their 50s and 60s which may explain our similar findings.

Generally, discordance can occur due to physiologic causes, pathophysiologic causes, anatomic causes, artifacts, and technical problems in measurement [5]. Depending on the population studied, some or all of these factors may be responsible for SHD. In interpreting DXA scan results, it is important to be aware of these factors, especially in the case of major discordance, when decisions for treatment need to be made. There are some limitations to this study. First, it is of a retrospective cross-sectional nature. Second, some aspects related to the possible role of obesity, such as diabetes mellitus, were not studied. Third, most patients were females which could reflect tendency to screen for osteoporosis in women rather than in men which was also noted in previous studies [17]. And fourth, the potential role of vitamin D deficiency - a common health problem in Saudi Arabia - was not studied.

## Conclusions

In conclusion, SHD is common among the elderly in Saudi Arabia. It is mainly caused by lower lumbar spine BMD. It is proposed that obesity may play a role in the occurrence of this discordance, either by altering measurements of lumbar spine BMD due to the presence of artifacts or increasing hip BMD through weight loading. Therefore, special attention should be paid when interpreting DXA results, especially in the case of obese patients.

## References

[1] Blake GM, Fogelman I (2009). The clinical role of dual energy x-ray absorptiometry. Eur J Radiol.

[2] Adams JE (2013). Advances in bone imaging for osteoporosis. Nat Rev Endocrinol.

[3] Kanis JA (1994). Assessment of fracture risk and its application to screening for postmenopausal osteoporosis. Osteoporos Int.

[4] Faulkner KG, von Stetten E, Miller P (1999). Discordance in patient classification using T-scores. J Clin Densitom.

[5] Woodson G (2000). Dual x-ray absorptiometry T-score concordance and discordance between the hip and spine measurement sites. J Clin Densitom.

[6] Chan CY, Subramaniam S, Mohamed N (2020). Prevalence and factors of T-score discordance between hip and spine among middle-aged and elderly Malaysians. Arch Osteoporos.

[7] Moayyeri A, Soltani A, Tabari NK, Sadatsafavi M, Hossein-Neghad A, Larijani B (2005). Discordance in diagnosis of osteoporosis using spine and hip bone densitometry. BMC Endocr Disord.

[8] Mounach A, Abayi DA, Ghazi M (2009). Discordance between hip and spine bone mineral density measurement using DXA: prevalence and risk factors. Semin Arthritis Rheum.

[9] Singh T, Ghosh A, Khandelwal N, Singla V, Gupta M (2020). Major and minor discordance in dual-energy x-ray absorptiometry diagnosis of osteoporosis - a cross-sectional, population-based, observational study in Indian women. J Midlife Health.

[10] Balachandran K, Mhadevan S, Asirvatham R (2020). Hip spine discordance in bone mineral density: prevalence and potential significance. Endocr Abstr.

[11] Hou J, He C, He W, Yang M, Luo X, Li C (2020). Obesity and bone health: a complex link. Front Cell Dev Biol.

[12] Ravn P, Cizza G, Bjarnason NH (1999). Low body mass index is an important risk factor for low bone mass and increased bone loss in early postmenopausal women. Early Postmenopausal Intervention Cohort (EPIC) study group. J Bone Miner Res.

[13] Felson DT, Zhang Y, Hannan MT, Anderson JJ (1993). Effects of weight and body mass index on bone mineral density in men and women: the Framingham study. J Bone Miner Res.

[14] Steinschneider M, Hagag P, Rapoport MJ, Weiss M (2003). Discordant effect of body mass index on bone mineral density and speed of sound. BMC Musculoskelet Disord.

[15] Patel R, Seah M, Blake GM, Jefferies AL, Crane FM, Fogelman I (1996). Concordance and precision of dual x-ray absorptiometry with a 10 s scan. Br J Radiol.

[16] Yoon BH, Kim DY (2021). Discordance between hip and spine bone mineral density: a point of care. J Bone Metab.

[17] Alswat K, Adler SM (2012). Gender differences in osteoporosis screening: retrospective analysis. Arch Osteoporos.

